# Emergency Colorectal Surgery in Those with Cirrhosis: A Population-based Study Assessing Practice Patterns, Outcomes and Predictors of Mortality

**DOI:** 10.1093/jcag/gwad040

**Published:** 2023-10-20

**Authors:** Lisa Zhang, Kelly Brennan, Jennifer A Flemming, Sulaiman Nanji, Maya Djerboua, Shaila J Merchant, Antonio Caycedo-Marulanda, Sunil V Patel

**Affiliations:** Department of Surgery, University of Ottawa, Ottawa, ON, Canada; Department of Surgery, Queen’s University, Kingston, ON, Canada; Department of Surgery, Queen’s University, Kingston, ON, Canada; Department of Surgery, Queen’s University, Kingston, ON, Canada; Department of Surgery, Queen’s University, Kingston, ON, Canada; Department of Surgery, Queen’s University, Kingston, ON, Canada; Orlando Health, Orlando, FL, USA; Department of Surgery, Queen’s University, Kingston, ON, Canada

**Keywords:** cirrhosis, colorectal surgery, emergency surgery, mortality, risk stratification

## Abstract

**Background:**

Those with cirrhosis who require emergency colorectal surgery are at risk for poor outcomes. Although risk predictions models exists, these tools are not specific to colorectal surgery, nor were they developed in a contemporary setting. Thus, the objective of this study was to assess the outcomes in this population and determine whether cirrhosis etiology and/or the Model for End Stage Liver Disease (MELD-Na) is associated with mortality.

**Methods:**

This population-based study included those with cirrhosis undergoing emergent colorectal surgery between 2009 and 2017. All eligible individuals in Ontario were identified using administrative databases. The primary outcome was 90-day mortality.

**Results:**

Nine hundred and twenty-seven individuals (57%) (male) were included. The most common cirrhosis etiology was non-alcoholic fatty liver disease (NAFLD) (50%) and alcohol related (32%). Overall 90-day mortality was 32%. Multivariable survival analysis demonstrated those with alcohol-related disease were at increased risk of 90-day mortality (hazards ratio [HR] 1.53, 95% confidence interval [CI] 1.2–2.0 vs. NAFLD [ref]). Surgery for colorectal cancer was associated with better survival (HR 0.27, 95%CI 0.16–0.47). In the subgroup analysis of those with an available MELD-Na score (*n* = 348/927, 38%), there was a strong association between increasing MELD-Na and mortality (score 20+ HR 6.6, 95%CI 3.9–10.9; score 10–19 HR 1.8, 95%CI 1.1–3.0; score <10 [ref]).

**Conclusion:**

Individuals with cirrhosis who require emergent colorectal surgery have a high risk of postoperative complications, including mortality. Increasing MELD-Na score is associated with mortality and can be used to risk stratify individuals.

## Introduction

Individuals with cirrhosis have higher post-operative morbidity and mortality following major abdominal surgery compared to those without cirrhosis. To quantify this added risk, observational studies and prediction models have been described; however, the majority were derived from historic cohorts and may not reflect the changing epidemiology of liver disease or advances in both the medical management of cirrhosis and perioperative practices in this high-risk group. Historically, risk of mortality in patients with cirrhosis has been estimated in clinical practice using either the Child-Turcotte-Pugh (CTP) Score^1,2^ or the Model of End Stage Liver Disease (MELD Score),^3^ which have also been shown to predict outcomes in patients with cirrhosis undergoing surgery. Neither take into consideration important clinical variables such as the type of surgery or urgency of surgery, which are both correlated with surgical outcomes. The Mayo risk score, which includes MELD plus the American Society of Anaesthesiologists (ASA) class and age, also predicts surgical mortality; however, its tendency to overestimate surgical risk has been criticized.^4,5^ Recently, the Veterans Outcomes and Costs Associated with Liver Disease (VOCAL)-Penn score was developed to stratify surgical risk for patients with cirrhosis based on broadly classified procedures.^4^ This scoring system allows for assessment of risk based on acuity of procedure (emergency vs. elective), but is limited in the broad classification of surgical procedures (i.e., “Abdominal Procedures”). Thus, there is no current risk stratification available for those specifically undergoing emergent colorectal procedures.

A number of previous studies^6–12^ have assessed mortality and morbidity in those with cirrhosis who underwent emergency colorectal surgery. These studies have reported 30-day mortality between 21% and 36% (compared with 9–15% in those without cirrhosis)^11–13^ and 30-day morbidity between 42% and 77% (compared with 15–35% in those without cirrhosis).^7,8,13,14^ There have been concerns raised regarding the generalizability of these results due to limitation in design (single centre and/or significant selection bias) or lack of inclusion of a contemporary cohort. In addition, a number of definitions of liver disease and cirrhosis were used, including the presence of ascites alone,^9^ partial criteria from the MELD or CTP scores,^13^ or confirmation by liver biopsy.^8,11^ Finally, several studies do not separate outcomes for emergent and elective colorectal surgery,^10–12^ which has been shown in previous literature to have a great impact on mortality outcomes for both patients with and without cirrhosis.^7,13^

Due to the limitations in the existing risk stratification tools and previous literature, the objective of this study was to use a large population cohort to describe practice patterns and short-term outcomes in patients with cirrhosis undergoing emergency major colorectal surgery. These results will be important for surgeons and hepatologists managing these complex individuals.

## Methodology

### Study design, setting, and participants

This is a population-based cohort study of individuals with cirrhosis undergoing emergent colorectal surgery in Ontario, Canada between 2009 and 2017. Ontario is Canada’s largest province (population 14.6 million) and provides single payer, universal health care through the Ontario Health Insurance Plan (OHIP). The study cohort was developed using provincial administrative data from ICES which, as an organization designated a prescribed entity under section 45 of Ontario’s Personal Health Information Privacy Act, houses and enables the use of healthcare data from Ontario residents without acquiring individual consent.

The administrative datasets used in this study included the OHIP physician claims database; the Canadian Institute for Health Information’s discharge abstract database (CIHI-DAD: information on inpatient admissions); the Registered Persons Database (RPDB: demographic information and vital status); the Postal Code Conversion Files (PCCF: postal code linkage to geographic areas and census area-based information, including income quintile); the Ontario Lab Information System (OLIS: pathology test results from hospital and clinical laboratories); Public Health Ontario (hepatitis data); the National Ambulatory Care Reporting System (NACRS: information on ambulatory care and emergency room visits); the Ontario Cancer Registry (OCR: information on cancer diagnosis); the Same Day Surgery database (SDS: information on day-surgery and outpatient clinic visits); the Ontario Ministry of Health and Long-term Care’s institution information system; and the ICES Physician Database (IPDB: information on physician demographics, specialty, and activity). Datasets used for this study were linked at the individual level using unique encrypted identifiers and analyzed at ICES Queen’s.

Individuals aged 18 years or older with cirrhosis were identified using a previously validated algorithm in ICES data.^15^ Those with a cirrhosis diagnosis before receiving a major emergency colorectal surgical procedure were included in the final study cohort. Major colorectal surgeries were identified using billing codes in the OHIP physician claims database and included colon or rectal resections and/or creation colostomy or ileostomy. Surgeries identified in OHIP were linked to their corresponding hospital admission record in CIHI-DAD. Only individuals with an emergency admission status indicated on their surgery record were included. Individuals were excluded if they did not have a unique identifier, did not have at least 2 years of OHIP eligibility prior to surgical admission date, received a liver transplant any time prior to surgery, or were missing a corresponding CIHI-DAD record for surgery admission.

### Variables, outcomes, and data sources

Patient demographics at the time of surgery including age, sex, and income quintile (a measure of relative household income within a census dissemination area, with quintile 1 being the lowest and quintile 5 being the highest) were identified from the RPDB and PCCF. Cirrhosis etiology was defined as either alcohol-associated disease (ALD), hepatitis C, hepatitis B, non-alcoholic fatty liver disease (NAFLD), or autoimmune liver disease from a hierarchical algorithm which incorporates both viral serology and diagnostic codes.^15^ A history of hepatic decompensation was identified using previously validated coding.^15^ In those with available data in OLIS, the MELD-Na score was calculated using the most recent lab values within one year of surgery for bilirubin, INR, creatinine, and sodium. The median time from the earliest laboratory test used to determine the MELD-Na score to time of surgery was 4 days (IQR 0–54 days). The Charlson co-morbidity index was calculated based on hospital admission diagnoses within 2 years prior to surgery. Patient co-morbidities of asthma, diabetes mellitus, hypertension, congestive heart failure, and chronic obstructive pulmonary disease were identified from ICES-validated cohorts.^16–19^ Patients with diagnostic codes for obesity within 2 years prior to surgery were also identified.

Acuity and details of the surgical procedures were derived from the corresponding hospital admission record from CIHI-DAD. The indications for surgery (colorectal cancer, inflammatory bowel disease [IBD], diverticulitis, other) were identified from the most responsible diagnosis during hospital admission, followed by additional diagnoses in CIHI-DAD or OHIP during hospital admission, and pre- and post-surgery diagnoses in CIHI-DAD, OHIP, NACRS, SDS, or OCR (for colorectal cancer) within 90 days of procedure. If the patient had no admitting, pre- or post-surgery diagnoses for colorectal cancer, inflammatory bowel disease, or diverticulitis, then the indication for surgery was classified as other. The hospitals where the surgeries took place were categorized as teaching or non-teaching hospitals according to the Ontario Ministry of Health and Long-term Care’s institution information system.

In-hospital and 90-day mortality were determined by linkage to the RPBD. Inpatient outcomes included intensive care unit (ICU) admission, length of ICU stay, and total hospital length of stay were identified from CIHI-DAD. Emergency department visits within 90 days post-discharge were identified from NACRS. Hospital re-admission within 90 days was determined from CIHI-DAD. Post-operative hepatic decompensation within 90 days after surgery was described using validated coding.^15^

### Statistical analysis

Patient demographics and surgery treatment details were described by 90-day mortality and short-term outcomes were described by MELD-Na score. Means with standard deviations were calculated for numeric variables, and count and proportions were tabulated for categorical variables. Statistical comparisons of means between groups were performed with one-way analysis of variance. Comparisons of proportions were made using the chi-square test and the Cochran-Armitage test for trend. Overall survival by MELD-Na score was evaluated with Kaplan Meier estimates. A priori identified risk factors associated with 90-day mortality were evaluated by multivariable Cox proportional hazards regression models. Patients were followed from time of surgery until the event of interest, death, occurred. Censoring was at 90 days post-surgery. Factors associated with mortality were included a priori and we calculated hazard ratios (HR) and 95% confidence intervals (CI) to quantify associations. We assessed the proportional hazards assumption for each model using Schoenfeld residuals and accounted for factors that violated the assumption by including an interaction term with time in the model.

As per ICES policy, data with small cells (<6) were not reported due to the risk of re-identification. Results were considered statistically significant at *P*-value <.05. All analyses were performed using SAS version 9.4 (SAS Institute, Cary, NC). The study was approved by the Research Ethics Board of Queen’s University (DMED-1651-13). In addition, this study was designed, analyzed, and reported in accordance with the STROBE (Strengthening the Reporting of Observational Studies in Epidemiology)^20^ and the RECORD (REporting of studies Conducted using Observational Routinely-collected health)^21^ statements.

## Results

**Figure 1.**
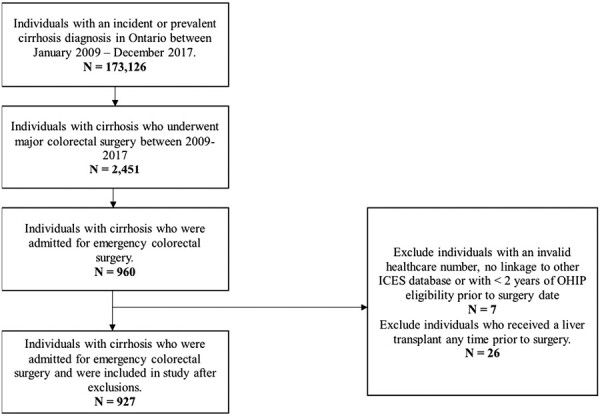
Flowsheet of included individuals.

A total of 173,126 individuals with cirrhosis were identified over the study period. Of these, 927 required emergency colorectal surgery (fig. 1).

### Patient demographics and cirrhosis details

Baseline demographics at the time of surgery are given in Table 1. The average age was 64 (SD 14.2), with the majority of individuals being male (*n* = 514, 55%). The lowest income quintiles were more commonly represented, while co-morbidities were common with the most frequent being hypertension (*n* = 598, 65%), chronic obstructive pulmonary disease (*n* = 342, 37%), and diabetes (*n* = 319, 34%). The most common etiology of cirrhosis was NAFLD (*n* = 465, 50%) followed by ALD (*n* = 292, 32%). Few individuals had a history of hepatic decompensation (*n* = 70, 8%). The MELD-Na score was available in 348 individuals (38%), with a median score of 12 (IQR 9–19).

**Table 1.** Baseline characteristics of those included in the cohort.

Variable	Alive at 90 days	Dead at 90 days	Total	*P*-value
	*N* = 634	*N* = 293	*N* = 927	
Mean age, years ± SD	62.10 ± 14.48	68.32 ± 12.71	64.07 ± 14.23	<.001
Median age (IQR)	63 (53–73)	70 (59–78)	65 (55–75)	
Female (%)	296 (46.69)	117 (39.93)	413 (44.55)	.500
Male (%)	338 (53.31)	176 (60.07)	514 (55.45)	
Income Quintile (%)				
Unknown	5–9 (0.79–1.41)	<6 (<2.04)	5–14 (0.53–1.51)	.020
1—lowest	154 (24.29)	95 (32.42)	249 (26.86)	
2	122–126 (19.24–19.87)	62–68 (21.16–23.21)	185–194 (19.96–20.92)	
3	133 (20.98)	43 (14.68)	176 (18.99)	
4	111 (17.51)	44 (15.02)	155 (16.72)	
5—highest	105 (16.56)	43 (14.68)	148 (15.97)	
Cirrhosis Etiology (%)				
Hepatitis B or C	65 (10.25)	39 (13.31)	104 (11.22)	.040
Autoimmune/Other	48 (7.57)	18 (6.14)	66 (7.12)	
Alcohol-related	186 (29.34)	106 (36.18)	292 (31.50)	
NAFLD	335 (52.84)	130 (44.37)	465 (50.16)	
History of decompensation (%)	30 (4.73)	40 (13.65)	70 (7.55)	
Mean MELD-NA ± SD	12.07 ± 5.71	18.68 ± 8.58	14.35 ± 7.51	.001
Median MELD-Na (IQR)	10 (8–16)	18 (11–25)	12 (9–19)	
Charlson Comorbidity Index (%)				
0	409 (64.51)	139 (47.44)	548 (59.12)	.001
1–3	165 (26.03)	91 (31.06)	256 (57.09)	
4+	60 (9.46)	63 (21.50)	123 (30.96)	

COPD, chronic obstructive pulmonary disease; MELD-Na, Model of End-Stage Liver Disease Score; NAFLD, non-alcoholic fatty liver disease.

### Treatment and hospital course

The indication for surgery was colorectal cancer in 35%, diverticulitis in 19%, inflammatory bowel disease 16%, and other/not classified 29%. The most common type of surgery was resection without anastomosis (40%), followed by resection with anastomosis (38%) (Table 2). The majority (585/927, 63%) required ICU admission with 186 (20%) being admitted to ICU prior to surgery, 301 (32%) admitted on the day of surgery, and 98 (11%) admitted after the surgical date. The median length of hospital stay was 16 days (IQR 9–32) (Table 2).

**Table 2.** Treatment characteristics of those included in the cohort.

Variable	Alive at 90 days	Dead at 90 days	Total	*P*-value
	*N* = 634	*N* = 293	*N* = 927	
Reason for surgery (%)				
Colorectal cancer	219 (34.54)	110 (37.54)	329 (35.49)	<.001
IBD	125 (19.72)	24 (8.19)	149 (16.07)	
Diverticulitis	134 (21.14)	45 (15.36)	179 (19.31)	
Other	156 (24.61)	114 (38.91)	270 (29.13)	
Type of surgery (%)				
Resection and anastomosis	266 (41.96)	84 (28.67)	350 (37.76)	<.001
Resection without anastomosis	232 (36.59)	141 (48.12)	373 (40.24)	
Resection, anastomosis and proximal diversion	31 (4.89)	21 (7.17)	52 (5.61)	
Colostomy/ileostomy only	105 (16.56)	47 (16.04)	152 (16.40)	
Hospital type (%)				
Non-teaching	375 (59.15)	180 (61.43)	555 (59.87)	.509
Teaching	259 (40.85)	113 (38.57)	372 (40.13)	
ICU admission (%)				
Before surgery	97 (15.30)	89 (30.38)	186 (20.06)	<.001
Day of surgery	177 (27.92)	124 (42.32)	301 (32.47)	
>1 day after surgery	57 (8.99)	41 (13.99)	98 (10.57)	
No ICU admission	303 (47.79)	39 (13.31)	342 (36.89)	

GI, Gastroenterology; IBD, inflammatory bowel disease; ICU, intensive care unit.

### Outcomes

The 90-day mortality of the cohort was 32% (*n* = 293) with in-hospital mortality being 25% (*n* = 233). In terms of cause of death at 90 days, 29 (3.1%) were liver-related, 66 (7.1%) were cancer-related, and other causes were responsible for 198 (21.4%). Hepatic decompensation within 90 days occurred in 17% of individuals (*n* = 159). Of the 694 individuals who were discharged from hospital, there was a high risk of emergency department visit (299/694, 43%) and readmission (244/694, 35%) within 90 days (Table 3).

**Table 3.** Short-term outcomes of the cohort following surgery.

Outcome	MELD-Na <10	MELD-Na 10-19	MELD-Na 20+	MELD-Na Missing	Total	*P*-value^a^
	N=133	N=135	N=80	N=579	N=927	
90-day mortality (%)	23 (17.29)	43 (31.85)	54 (67.50)	173 (29.88)	293 (31.61)	<.001
In-hospital mortality (%)	13 (9.77)	31 (22.96)	52 (65.00)	137 (23.66)	233 (25.13)	<.001
ICU admission (%)	62 (46.62)	95 (70.37)	73 (91.25)	355 (61.31)	585 (63.11)	<.001
Mean hospital length of stay, days ± SD	24.69 ± 29.10	31.80 ± 45.19	30.44 ± 39.26	27.30 ± 34.53	27.85 ± 36.01	<.001
Median hospital length of stay, days (IQR)	17 (9-27)	19 (9-35)	16 (8-32)	16 (8-31)	16 (9-32)	
90-day hepatic decompensation (%)	15 (11.28)	35 (25.93)	20 (25.00)	89 (15.37)	159 (17.15)	.008
90-day readmission (%)^b^	45/120 (37.50)	44/104 (42.31)	13/28 (46.43)	142/442 (32.13)	244/694 (35.16)	.610
90-day emergency department visit (%)^b^	57/120 (47.50)	44/104 (42.31)	10/28 (35.71)	188/442 (42.53)	299/694 (43.08)	.470

ICU, intensive care unit; IQR, interquartile range; MELD-Na, Model for end stage liver disease; SD, standard deviation.

^a^Statistical testing compared those with an available MELD-Na only (excluded those missing a MELD-Na).

^b^Only those discharged from hospital included.

Those who died within 90 days were older (mean age 68.3 [STD 12.7] vs. 62.1 [STD 14.5], *P* < .001) and had increasing Charlson Co-Morbidity score (*P* < .001). Cirrhosis etiology was also associated with 90-day mortality, with the highest risk in those with alcohol related liver disease (36.3% vs. NAFLD 28.0% vs. Other 33.5%, *P* = .04) (Table 1).

Cox Proportional Hazards analysis is reported in Table 4. Increasing age (HR 1.03, 95%CI [per year]), and increasing Charlson Co-Morbidity Score (Score 4+ HR 2.33, 95%CI 1.72–3.14; Score 1–3 HR 1.34, 95%CI 1.03–1.75; Score 0 [ref]) were associated with mortality. Cirrhosis etiology was also associated with mortality, were those with alcoholic related having the highest hazards of death (HR 1.53, 95%CI 1.17–2.00) followed by “other” (HR 1.40, 95%CI 1.01–1.93), with NAFLD as the referent. Colorectal cancer was protective when compared with other diagnoses (HR 0.27, 95%CI 0.16–0.47).

**Table 4.** Cox-proportional hazards analysis of mortality and risk factors.

		Univariable		Multivariable	
Variable		HR (95% CI)	*P*-value	HR (95% CI)	*P*-value
Age at time of surgery, per year		1.03 (1.02–1.04)	<.001	1.03 (1.02–1.04)	<.001
Sex	Male	1.27 (1.01–1.60)	.045	1.22 (0.96–1.55)	.106
	Female	1.00 (ref)	.	1.00 (ref)	.
Cirrhosis etiology	Alcohol-related	1.42 (1.10–1.84)	.007	1.53 (1.17–2.00)	.002
	Other	1.25 (0.91–1.70)	.167	1.40 (1.01–1.93)	.044
	NAFLD	1.00 (ref)	.	1.00 (ref)	.
Charlson comorbidity index	1–3	1.48 (1.14–1.93)	.004	1.34 (1.03–1.75)	.032
	4+	2.44 (1.81–3.29)	<.001	2.33 (1.72–3.14)	<.001
	0	1.00 (ref)	.	1.00 (ref)	.
Reason for surgery*	Colorectal cancer	0.37 (0.21–0.64)	<.001	0.27 (0.16–0.47)	<.001
	IBD, diverticulitis or other	1.00 (ref)	.	1.00 (ref)	.
Interaction term		1.55 (1.27–1.88)	<.001	1.52 (1.25–1.85)	<.001

CI, confidence interval; HR, hazards ratio; IBD, inflammatory bowel disease; NAFLD, non-alcoholic fatty liver disease.

*Variable violates the proportional hazards assumption. An interaction term of the variable with log of time was included in the model to address the violation.

### Model for end-stage liver disease and outcomes

Subgroup analyses were completed in those with a MELD-Na score available (*n* = 348/927, 38%). A comparison of those with and without a MELD-Na score available is presented in Supplementary Table 1. Although some difference between the groups existed, the groups were similar in terms of their baseline characteristics.

Outcomes by MELD-Na score are reported in Table 3. The risk of death at 90 days increased from 17% in those with a MELD-Na of <10 to 68% in those with a MELD-Na of 20+. ICU admission was much more common in those with a high MELD-Na (91%) compared with those with a low MELD-Na (47%). Other 90-day outcomes such as hepatic decompensation, readmission to hospital, and ED visits did not demonstrate a similar relationship.

**Figure 2.**
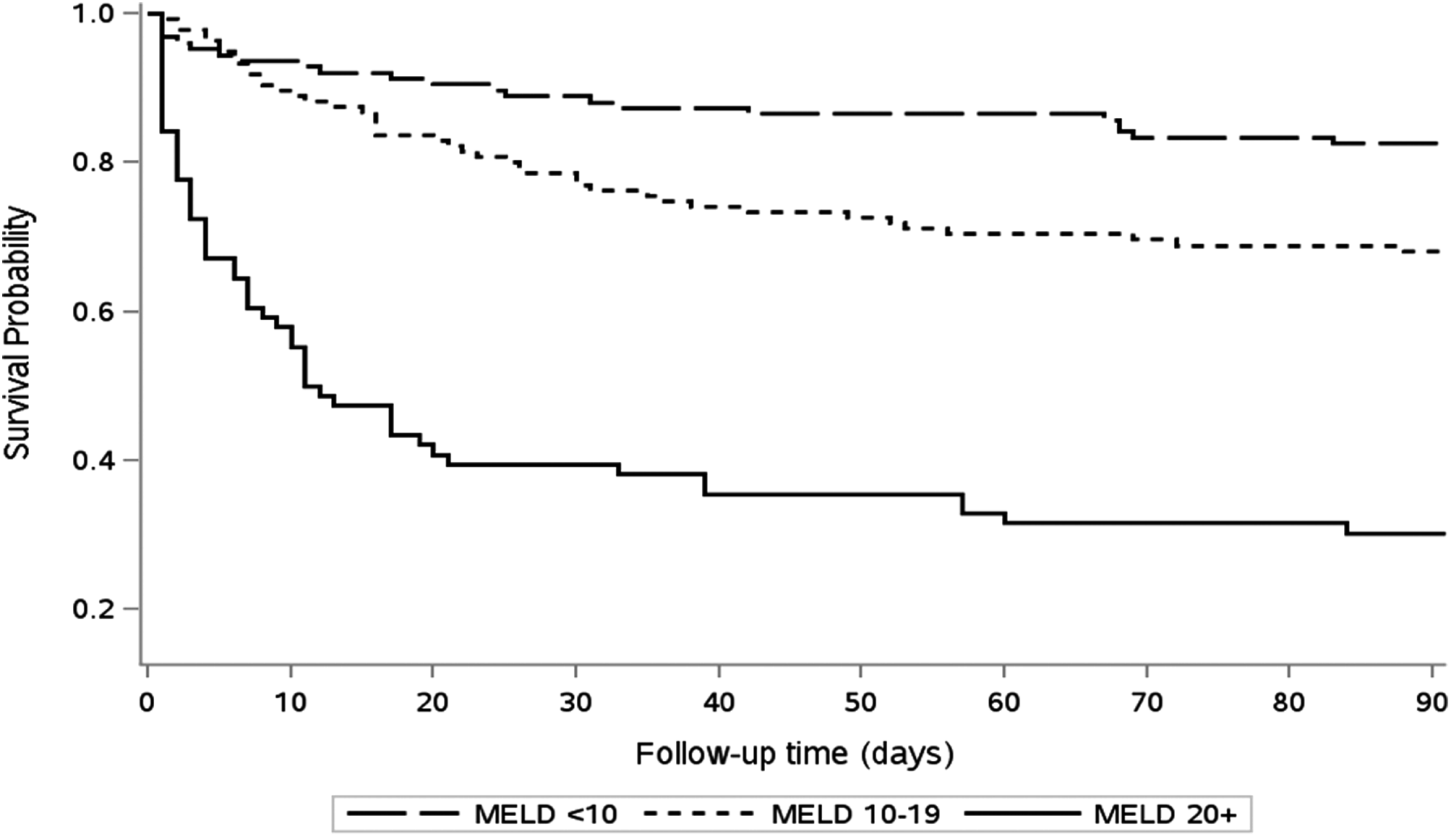
Kaplan Meier Survival analysis by MELD-Na (model for end-stage liver disease).

Survival analysis was completed using both a Kaplan Meier Analysis (fig. 2) and Cox Proportional Hazards Analysis (Table 5). Multivariable analysis demonstrated an association between MELD-Na score and mortality (MELD-Na 20+ HR 6.56, 95%CI 3.94–10.9; MELD-Na 10–19 HR 1.97, 95%CI 1.19–3.27; MELD-Na <10 [ref]).

**Table 5.** Cox-proportional HR of mortality, in those with an available MELD-Na (model for end-stage liver disease) score (*n* = 348/927, 38%).

		Univariable		Multivariable	
Variable		HR (95% CI)	*P*-value	HR (95% CI)	*P*-value
Age at time of surgery, per year		1.04 (1.02–1.05)	<.001	1.04 (1.03–1.06)	<.001
Sex	Male	1.23 (0.85–1.78)	.273	1.18 (0.80–1.74)	.413
	Female	1.00 (ref)	.	1.00 (ref)	.
Cirrhosis etiology	Alcohol-related	1.14 (0.76–1.72)	.530	1.41 (0.90–2.21)	.132
	Other	0.96 (0.61–1.53)	.871	1.10 (0.68–1.80)	.694
	NAFLD	1.00 (ref)	.	1.00 (ref)	.
Charlson comorbidity index	1–3	1.32 (0.88–1.99)	.182	1.09 (0.72–1.65)	.676
	4+	1.76 (1.10–2.81)	.018	1.39 (0.86–2.25)	.175
	0	1.00 (ref)	.	1.00 (ref)	.
Reason for surgery*	Colorectal cancer	0.26 (0.10–0.65)	.004	0.25 (0.10–0.64)	.004
	IBD, diverticulitis or other	1.00 (ref)	.	1.00 (ref)	.
MELD-Na score	10-19	1.97 (1.19–3.27)	.009	1.79 (1.07–2.99)	.027
	20+	6.35 (3.89–10.38)	<.001	6.56 (3.94–10.90)	<.001
	<10	1.00 (ref)	.	1.00 (ref)	.
Interaction term		1.79 (1.28–2.48)	<.001	1.66 (1.19–2.32)	.003

CI, confidence interval; HR, hazards ratio; IBD, inflammatory bowel disease; NAFLD, non-alcoholic fatty liver disease; ref, Referent.

*Variable violates the proportional hazards assumption. An interaction term of the variable with log of time was included in the

model to address the violation.

## Discussion

This study reports practice patterns and short-term outcomes for individuals with cirrhosis who required emergent colorectal surgery. We demonstrated that there was a high risk of 90-day and in-hospital mortality (32% and 25%, respectively). In addition, there was a high risk of 90-day hepatic decompensation (17%) and ICU admission (63%). Of those individuals who survived their initial surgical admission, a significant number subsequently visited the ED (43%) or were readmitted to hospital (35%) within 90 days of surgery.

In addition, we demonstrated that MELD-Na score was highly predictive of poor outcomes. In fact, those with a MELD-Na score of >20 had a 67% 90-day mortality, and a 91% risk of ICU admission. The association between high MELD-Na score and death was demonstrated in the multivariable analysis as well. In the minority that were able to be discharged, nearly half were readmitted within 90 days.

### Strengths and limitations

Our study is a large population-based study. For this reason, there are a number of strengths that improve the applicability and generalizability of the study. First, we used a validated algorithm to identify those diagnosed with cirrhosis in the province. In addition, we used valid methods of identifying both the indication for surgery (i.e., colorectal pathology) and acuity of surgery (emergency surgery). We included all eligible patients, reducing the risk of selection bias and misclassification of the diagnosis and treatment. As the data for this study was ascertained using linked administrative databases within a single-payer universal health care system, loss to follow up was minimal. Our selected primary outcome (mortality) is a strong binary outcome with limited risk of misclassification. Finally, we were able to include a contemporary cohort of individuals who presumably benefitted from modern management of cirrhosis and modern perioperative care.

Despite these strengths, several well-described limitations of population-based studies exist within this study.^22^ Pertinent to the current study, missing data was an issue. All data elements necessary for MELD-Na score calculation was available in <50% of individuals. In comparing baseline characteristics and outcomes (Supplementary Table 1), we found those with MELD-Na score available and those without to be comparable. Despite this, important poorly captured differences between those with and without a MELD-Na score may exist. In addition, due to the nature of this study, specific details regarding diagnoses and treatment are not known. For instance, a number of individuals had “other” as a reason for surgery. Generally this included “bleeding,” “perforation,” or “obstruction” without a specific diagnosis (i.e., colorectal cancer vs. diverticular disease). Similarly in those with a diagnosis (i.e., colorectal cancer, diverticular disease, IBD), the specific indication was unknown (i.e., bleeding vs. perforation vs. obstruction). The difference in 90-day mortality between different etiologies of liver disease should also be interpreted with some caution, as the numbers in the “other” and “alcohol-related liver disease” categories were small relative to NAFLD. It is important to note, that only those undergoing surgery were included in this cohort. We were unable to capture or assess those who may have had an indication for surgery, but ultimately did not undergo surgical management. These individuals would presumably be those with more severe liver disease (i.e., MELD-Na > 20) or more significant co-morbidities. Thus, our analyses likely underestimate the true surgical risk of these individuals.

Finally, due to the nature of the study design (retrospective cohort) and completeness of the data sources (i.e., linked administrative databases), there is likely unresolved confounding in our adjusted analysis.

### Comparison with previous studies

The MELD-Na score has previously been used to predict mortality after major surgery in patients with cirrhosis, with a reported 30-day mortality ranging from 6% to 10% (score <11) to 25–44% (score 12–20) to >50% (score >20).^5^ In our cohort, we identified a similar trend in both in-hospital and 90-day mortality, respectively: 10% and 17% (score < 10), 23% and 32% (score 10–19), and 65% and 68% (score > 20). As for the Child-Pugh score, the reported mortality is 10% for Child Class A, 30% for Child Class B, and 75% for Child Class C patients undergoing abdominal surgery.^23^ There is no universally accepted conversion between this score and the MELD-Na score, so we are unable to directly compare the Child-Pugh Classification mortalities to our own MELD-Na categories. A previous study did compare Child-Pugh Class C with a MELD-Na score of >14, and found that the MELD-Na score was a better clinical predictor for mortality.^24^

In terms of emergency colorectal surgery specific results, we report similar mortality risk (current study: in-hospital 25%, 90-day 32%) as a number of previous studies (30-day mortality between 21% and 36%).^6,7,13^ Similarly, we report high rates of morbidity (hepatic decompensation 17%; readmission 35%; ED visit 43%), which is consistent with some previous reports.^7,8,13,14^

The VOCAL-Penn study suggested NAFLD as the liver disease etiology with the highest mortality, while our results show that alcohol-related liver disease was associated with a higher hazard of death. We know that alcohol is a risk factor for developing several extrahepatic cancers, including colorectal cancer.^25^ However, a systematic review has also identified an association between NAFLD and colorectal cancer development.^26^ In our cohort, colorectal cancer appeared to be protective compared to other indications for surgery. The complex etiology and pathway to colorectal cancer, and its relationship to underlying liver disease, continue to be areas that require further study.

## Conclusion

In conclusion, we found that those with liver cirrhosis who required emergency colorectal surgery had a high risk of mortality and postoperative complications. We also found that increasing severity of liver disease (as determined using MELD-Na) was associated with worse outcomes, most notably a very high risk of mortality. Other complications, such as hepatic decompensation, intensive care unit admission, emergency department admission, and re-admission to hospital were common. This group continues to have poor outcomes despite modern medical, surgical, and perioperative care.

## Supplementary data

Supplementary data are available at *Journal of the Canadian Association of Gastroenterology* online.

gwad040_suppl_Supplementary_Materials

## Data Availability

The dataset from this study is held securely in coded form at ICES. While legal data sharing agreements between ICES and data providers (e.g., healthcare organizations and government) prohibit ICES from making the dataset publicly available, access may be granted to those who meet pre-specified criteria for confidential access, available at www.ices.on.ca/DAS (email: das@ices.on.ca). The full dataset creation plan and underlying analytic code are available from the authors upon request, understanding that the computer programs may rely upon coding templates or macros that are unique to ICES and are therefore either inaccessible or may require modification.

## Notes

This work was presented as a Podium Presentation at the American College of Surgeons Clinical Congress in October 2021 (virtual).
