# Supplementary material for: Emergency Colorectal Surgery in Those with Cirrhosis: A Population-based Study Assessing Practice Patterns, Outcomes and Predictors of Mortality
Source: J Can Assoc Gastroenterol. 2023 Oct 20;7(2):160–8. doi: 10.1093/jcag/gwad040 (PMC10999774; doi:10.1093/jcag/gwad040)
Supplement: gwad040_suppl_Supplementary_Materials [file gwad040_suppl_supplementary_materials.zip › gwad040_suppl_Supplementary_Table_1.docx]

Supplemental Table 1: Comparison of those with and without a MELD- Na (model for end stage liver disease) score available

| **Variable** | **MELD score available** | **No MELD score** | **p-value** |
| --- | --- | --- | --- |
|  | N=348 | N=579 |  |
|  | No (%) | No (%) |  |
| Age (in years) |  |  |  |
| Mean ± SD | 62.97 ± 14.66 | 64.73 ± 13.94 | 0.069 |
| Median (IQR) | 64 (54-74) | 66 (55-75) | 0.147 |
| Age groups |  |  | 0.288 |
| <40 | 27 (7.76) | 24 (4.15) |  |
| 40-49 | 30 (8.62) | 53 (9.15) |  |
| 50-59 | 75 (21.55) | 132 (22.80) |  |
| 60-69 | 86 (24.71) | 136 (23.49) |  |
| 70-79 | 85 (24.43) | 148 (25.56) |  |
| 80+ | 45 (12.93) | 86 (14.85) |  |
| Sex |  |  | 0.272 |
| Female | 147 (42.24) | 266 (45.94) |  |
| Male | 201 (57.76) | 313 (54.06) |  |
| Income quintile* |  |  | 0.291 |
| 1 - lowest | 84 (24.14) | 178 (30.74) |  |
| 2 | 76 (21.84) | 110 (19.00) |  |
| 3 | 70 (20.11) | 106 (18.31) |  |
| 4 | 59 (16.95) | 96 (16.58) |  |
| 5 - highest | 59 (16.95) | 89 (15.37) |  |
| Cirrhosis etiology |  |  | 0.008 |
| Hepatitis C | 41 (11.78) | 46 (7.94) |  |
| Hepatitis B | 11 (3.16) | 6 (1.04) |  |
| Autoimmune/Other | 31 (8.91) | 35 (6.04) |  |
| Alcohol-related | 107 (30.75) | 185 (31.95) |  |
| NAFLD | 158 (45.40) | 307 (53.02) |  |
| History of decompensation | 36 (10.34) | 34 (5.87) | 0.013 |
| Charlson Comorbidity Index |  |  | <0.001 |
| 0 | 175 (50.29) | 373 (64.42) |  |
| 1 | 40 (11.49) | 49 (8.46) |  |
| 2 | 29 (8.33) | 51 (8.81) |  |
| 3 | 45 (12.93) | 42 (7.25) |  |
| 4 | 18 (5.17) | 21 (3.63) |  |
| 5+ | 41 (11.78) | 43 (7.43) |  |
| Asthma | 59 (16.95) | 103 (17.79) | 0.746 |
| Diabetes Mellitus | 134 (38.51) | 185 (31.95) | 0.042 |
| Hypertension | 222 (63.79) | 376 (64.94) | 0.724 |
| Congestive heart failure | 78 (22.41) | 109 (18.83) | 0.187 |
| COPD | 130 (37.36) | 212 (36.61) | 0.821 |
| Obesity | 12 (3.45) | 24 (4.15) | 0.595 |
| In-hospital mortality | 96 (27.59) | 137 (23.66) | 0.182 |
| 90-day mortality | 120 (34.48) | 173 (29.88) | 0.144 |
| Hepatic decompensation within 90 days after surgery | 70 (20.11) | 89 (15.37) | 0.064 |
| Hospital readmission within 90 days after surgery | 102 (29.31) | 142 (24.53) | 0.109 |
| ED visit within 90 days after surgery | 111 (31.90) | 188 (32.47) | 0.857 |

*<2% of patients missing information on income quintile. Patients with missing income quintile were grouped with quintile 1.
